# Female Sexual Function of Overweight Women with Gestational Diabetes Mellitus – A Cross-Sectional Study

**DOI:** 10.1371/journal.pone.0095094

**Published:** 2014-04-15

**Authors:** Meireluci Costa Ribeiro, Mary Uchiyama Nakamura, Maria Regina Torloni, Marco de Tubino Scanavino, Flávia Burin Scomparini, Rosiane Mattar

**Affiliations:** 1 Department of Obstetrics, São Paulo Federal University (UNIFESP), São Paulo, Brazil; 2 Internal Medicine Department, São Paulo Federal University (UNIFESP), São Paulo, Brazil; 3 Department and Institute of Psychiatry, São Paulo State University Medical School (FMUSP), São Paulo, Brazil; University of Tennessee Health Science Center, United States of America

## Abstract

Obesity and gestational diabetes mellitus (GDM) are increasing worldwide and may compromise female sexual function. We hypothesize that among GDM patients in the third trimester of pregnancy, those with excess body fat would have worse female sexual function scores than normal weight women. Our aim was to assess the sexual function of overweight compared to normal weight women with GDM. This was a cross-sectional survey involving 143 Brazilian women with GDM in the third trimester of pregnancy: 76 were overweight (pre-pregnancy body mass index-BMI≥25.0 Kg/m^2^) and 67 were normal weight (BMI 18.5–24.9 Kg/m^2^). Participants were recruited from March 2010 to April 2013 at the antenatal clinic of a single public tertiary teaching institution. The Female Sexual Function Index (FSFI) questionnaire was used to assess sexual function. Overall, 51.7% of the 143 participants were at risk for sexual dysfunction symptoms (FSFI scores ≤26); this rate was significantly higher among overweight compared to normal weight women (60.5% versus 41.8%, p = 0.038). Mean total FSFI scores were significantly lower in overweight compared to normal weight women (21.7±9.2 versus 24.9±8.0, p = 0.029). Compared to normal weight women, overweight participants had lower mean scores in desire (3.4±1.2 versus 4.0±1.4, p = 0.007) and lubrication (3.8±2.0 versus 4.5±1.6, p = 0.023). According to these results, overweight women with GDM in the third trimester of pregnancy have lower female sexual function scores than normal weight women with the same disorder.

## Introduction

Sexual dysfunction symptoms are frequent during pregnancy. They are attributed to physical [1], [2], [3], psychological [4], relationship [5], [6], sociocultural and religious [6], [7] factors, as well as to the common fears and myths about potential harms of sexual intercourse on pregnancy or the fetus [3], [7], [8], [9], [10], [11]. All phases of the female sexual response cycle, including desire, arousal and orgasm [12] can be compromised in pregnant women, especially during the third trimester of pregnancy [8], [13], [14], [15].

Gestational diabetes mellitus (GDM) is defined as glucose intolerance that begins during pregnancy [16]. It affects from 1% to 14% of all pregnant women depending on the diagnostic criteria used and population characteristics and it is the most frequent endocrine disorder of pregnancy [17]. Due to the increasing prevalence of obesity, one of the main risk factors for GDM [18], [19], the prevalence of GDM is also expected to increase over the next years, both in developed and developing countries [20]. Recent studies [21], [22], [23] have investigated the prevalence of sexual dysfunction symptoms in women with GDM. Investigators hypothesized that the many adjustments associated with the diagnosis of GDM, as well as the added stress related to increased risks of maternal and perinatal complications, could affect the sexual function of these women [24]. While some studies confirmed this hypothesis [23], others did not identify significant differences in sexual function scores of women with and without GDM in the third trimester of pregnancy [21], [22]. These controversial findings could in part be due to differences in population characteristics, including the prevalence of obese women in these studies.

Obesity has been shown to compromise the sexual function of reproductive age women [25], . Obese pregnant women are at higher risk for depression and anxiety [29],[30],[31], and lower quality of life [32], [33], factors that increase the likelihood for sexual dysfunction symptoms [34]. According to the latest national demographic survey, 60% of reproductive age women (15–49 years) in Brazil are overweight (body mass index - BMI>24.9 Kg/m) and one third of these women are obese (BMI≥30 Kg/m ^2^) [35].

There are studies on the sexual function of women with GDM and of obese pregnant and non-pregnant women [21], [22], [23], [25], [26], [27], [36]. However, to the best of our knowledge, there are no previous studies which investigated the sexual function of obese women with GDM. We hypothesize that among GDM patients in the third trimester of pregnancy, those with excess body fat would have worse female sexual function scores than those of normal body weight.

### Aim

To assess and compare the sexual function of third trimester overweight and normal weight women with GDM.

## Material and Methods

### Design and Data Collection

This was a cross-sectional study involving women with a diagnosis of GDM managed at a large public university setting in São Paulo, Brazil, during a three-year period (2010–2013). As part of their routine antenatal care and following international recommendations [16], all women managed in the antenatal care clinics of this university undergo a 75 g 2-hours oral glucose tolerance test (OGTT) between 24 and 28 weeks of gestation to diagnose GDM. Pregnant women with normal results (fasting <92 mg/dL, 1 hour post-load <180 mg/dL and 2 hour post-load <153 mg/dL) continue to be managed in their usual antenatal care clinics. Those with at least one abnormal glucose value (equal or greater than the aforementioned levels) receive a diagnosis of GDM [37] and are referred to a specialized diabetes antenatal care clinic within the university where they are managed by a multi-professional team (obstetrician, endocrinologist, nurses, dietitian, psychologist and physical therapist) until delivery. At this specialized clinic, all women with a diagnosis of GDM receive the same type of care, regardless of their BMI. Women with normal OGTT results continue being managed at the regular antenatal care unit, in another building.

Potential participants were approached while waiting for their routine visits at this diabetes clinic. Inclusion criteria were: 20 years of age or older, singleton pregnancy, gestational age 28 weeks or more (based on ultrasound), diagnosed with GDM at least 4 weeks before and pre-pregnancy BMI≥18.5 Kg/m^2^ (based on their self-reported pre-pregnancy weight and measured height). Only women who were in a stable relationship with the same man for at least six months were eligible to participate. Those in sexual abstinence due to a medical recommendation (e.g. to treat vaginal infections or placenta previa, premature rupture of membranes, threatened preterm labor) were not eligible. Women taking antihypertensive medication or medications that can interfere with sexual function (such as antidepressants, tranquillizers, or hormones) were also excluded, as well as those with a current or previous history of psychiatric disorder (e.g. depression, schizophrenia, and neuroses), substance abuse (alcohol or illicit drugs), pregnancy resulting from rape, admission to the hospital in the last 30 days and absent or sexually unavailable partner in the last month. Based on their prepregnancy BMI, the participants were divided in two groups: normal weight (BMI 18.5–24.9 kg/m^2^) and overweight (BMI (≥25 Kg/m^2^) [38]. The health personnel caring for these women in the specialized antenatal care unit were blinded to the results of the questionnaires.

### Ethics Statement

All participants gave written informed consent S1 and the study was approved by the Ethics Committee of São Paulo Federal University (Process n. 0281/10).

### Details of the Questionnaires

Participants were asked to fill individual, anonymous, written questionnaires. Information collected included age, self-reported race, education, marital status, religion, occupation, family income, parity, family planning, gestational age and pre-pregnancy BMI. Information regarding the date of GDM diagnosis, current gestational age, use of insulin and their most recent HbA1c level were collected from their antenatal charts. Gestational age was based on menstrual dates confirmed through obstetric ultrasounds.

All participants were asked to answer the Brazilian version of the Female Sexual Function Index (FSFI), a self-responsive questionnaire, with 19 questions [39]. This questionnaire is accepted as a valid tool to measure female sexual function, has good internal consistency (Cronbach's alpha 0.791 to 0.914) and evaluates all phases of the female sexual cycle (desire, arousal, and orgasm), as well as sexual satisfaction, and dyspareunia in the last four weeks [39], [40]. Individual scores for each question range from a minimum of 0 or 1 to a maximum of 5. Domain scores are obtained by adding individual question scores and multiplying these by a specific factor. The full scale score is obtained by adding the six domain scores and ranges from 2 to 36, with higher scores reflecting a better sexual function. Women with a total score ≤26 are classified as being at risk for sexual dysfunction [41]. Only the desire domain has an established cutoff; it is scored on a 10-point scale, with final score ≤5 indicating sexual desire dysfunction symptoms (according to personal communication to the main author). This questionnaire has been extensively used to assess sexual function in pregnancy [8], [15], [22], [42], [43], [44]. Each participant took an average of 15 minutes to fill the questionnaires and those who did not answer all questions of the FSFI were excluded from the study.

### Statistical Aspects

Based on the 59% prevalence of sexual dysfunction symptoms among adult Brazilian women with GDM in the third trimester of pregnancy [22] and assuming that overweight women would have a 40% higher prevalence of this disorder, with an α = 0.05 and a β = 90%, the study would need to recruit 62 participants in each group (normal and overweight). Participant characteristics are presented descriptively and compared between the two groups using two-tailed Student's *t* or Chi-square tests. Differences in mean FSFI scores between the two groups were assessed using Student's *t* test. P values p<0.05 were considered significant. Statistical analyses were performed using InStat 3 (Statistical Services Centre, University of Reading, UK).

### Main Outcome Measures

Main outcomes were mean total FSFI scores, mean individual scores of each FSFI domain and the prevalence of patients with low sexual function scores (FSFI≤26) in the two groups (normal and overweight).

## Results

A total of 155 women were invited to participate, 12 were excluded (four declined, three were using antihypertensive medications, two were in sexual abstinence during the last month because their husbands did not want to engage in sexual activities; three were in sexual abstinence due to medical recommendations) resulting in 143 participants included in the final analyses: 67 normal weight and 76 overweight. Within the overweight group, there were 30 pre-obese (BMI≥25–29.9 Kg/m^2^) and 46 obese (BMI≥30 Kg/m^2^) women. Since mean total FSFI scores of these two subgroups did not differ significantly (21.3±9.4 and 21.9±9.2, respectively, p = 0.784), they were combined in a single “overweight” group for further analyses.

The main characteristics of the participants were similar among normal and overweight women (Table 1). Most were married, catholic, multipara, employed, had at least 9 years of formal education, a mean monthly family income between 340–1020 US dollars and reported that their pregnancy had not been planned. Over 75% of the participants were being treated only through diet at the moment of the survey.

**Table 1:** Main characteristics of 143 women with GDM in the third trimester of pregnancy.

Variable	Normal weight BMI 18.5–24.9 Kg/m^2^ (n = 67)	Overweight group BMI≥25 Kg/m^2^ (n = 76)	*P* value
Age, years	32.2±6.6	33.4±5.0	0.219*
Gestational age, weeks	32.8±3.4	32.5±3.2	0.588*
Prepregnancy BMI, Kg/m^2^	22.4±2.4	31.1±3.9	0.0001*
Time since diagnosis of GDM, weeks	8.9±4.5	9.6±4.8	0.372*
Marital Status			
Married**	63 (94.0)	71 (93.4)	
Single or divorced	4 (6.0)	5 (6.6)	0.100+
Religion			
Catholic	33 (49.3)	46 (60.5)	
Protestant	23 (34.3)	18 (23.7)	
Other/none	11 (16.4)	12 (15.8)	0.327+
Education, years			
<9	11 (16.4)	18 (23.7)	
9 to 12	46 (68.7)	50 (65.8)	
>12	10 (14.9)	8 (10.5)	0.468+
Race			
White	20 (29.9)	34 (44.8)	
Black	9 (13.4)	11 (14.5)	
Mixed	38 (56.7)	31 (40.1)	0.136+
Employment			
Housewife	18 (26.9)	28 (36.8)	
Employed	49 (73.1)	48 (63.2)	0.203+
Family income± (minimum wage per month)			
<3	2 (3.0)	3 (3.9)	
1 a 3	43 (64.2)	42 (55.3)	
>3	22 (32.8)	31 (40.1)	0.555+
Parity			
0	25 (37.3)	18 (23.7)	
1	20 (29.9)	30 (39.4)	
2 or more	22 (32.8)	28 (36.8)	0.192+
Unplanned pregnancy	46 (68.7)	56 (73.4)	0.633+
Currently using insulin	14 (20.9)	18 (23.7)	0.843+

**Or common law marriage.

*Student's *t*-test.

+ Chi-square test.

± One minimum wage represents approximately US$302,67.

Values presented as N (%) or mean (Standard Deviation [SD]).

GDM =  gestational diabetes mellitus.

The mean total FSFI score of the 143 GDM was 23.3±8.6. Over half of the participants (n = 74) women scored ≤26 in the FSFI questionnaire and were therefore classified as being at risk for sexual dysfunction symptoms (Table 2). A total of 46 overweight women scored ≤26 on the FSFI compared to 28 of the normal weight women (60.5% versus 41.8%, respectively p = 0.038) (Table 2). Mean desire domain scores were significantly lower in overweight compared to normal weight patients (3.4±1.2 versus 4.0±1.4, p = 0.007). The proportion of overweight women reporting problems in desire (domain score ≤5) was significantly higher than normal weight women: 48.7% versus 28.3%, respectively (p = 0.021). Overweight women also had significantly lower scores for the lubrication domain, compared to normal weight women (3.8±2.0 versus 4.5±1.6, respectively p = 0.023) (Table 2). The complete dataset with detailed information of all 143 participants is available upon request.

**Table 2:** Sexual function scores^1^ of 143 women with GDM in the third trimester of pregnancy, according to prepregnancy body mass index.

	Normal weight BMI 18.5–24.9 (n = 67)	Overweight BMI≥25 (n = 76)	*P* value
Women with scores ≤26	28 (41.8)	46 (60.5)	0.038**
Mean total score□	24.9±8.0	21.7±9.2	0.029*
Domains			
Desire^+^	4.0±1.4	3.4±1.2	0.007*
Arousal^±^	3.9±1.4	3.4±1.6	0.052*
Lubrication^±^	4.5±1.6	3.8±2.0	0.023*
Orgasm^±^	4.0±1.8	3.6±2.0	0.210*
Satisfaction×	4.4±1.7	3.9±2.1	0.118*
Dyspareunia^±^	4.1±2.0	3.7±2.1	0.251*

1. Scores obtained on the Female Sexual Function Index questionnaire [40].

□ Scores range from 2 to 36; total score ≤26 suggest risk for sexual dysfunction [41].

** Chi-square test.

* Student's *t*-test.

+ Scores range from 1.2 to 6.

±Scores range from 0 to 6.

× Scores range from 0.8 to 6.

Values presented as N (%) or mean (Standard Deviation [SD]).

## Discussion

The mean total FSFI scores of our 143 GDM patients were low. This finding has been previously reported in studies with GDM patients [21], [22], [23] and is also common among healthy pregnant women in the third trimester of pregnancy [8], [13], [14], [15]. Over 50% of all women in this survey scored below the cutoff used to classify women as being at risk for sexual dysfunction. Similarly, Souza *et al*. [23] reported that 67% of his 33 GDM patients were at risk for sexual dysfunction and had significantly lower mean total FSFI scores than their 88 healthy pregnant women (23.6 vs. 26.5, p = 0.03, respectively). However these authors included only women in the second trimester of pregnancy (20 to 25 weeks) and did not evaluate the effect of maternal adiposity on these indices.

As hypothesized, overweight GDM patients had a more compromised sexual function than normal weight GDM patients, with significantly lower mean total FSFI scores and a higher prevalence of participants with sexual dysfunction symptoms. Although overweight women scored lower in all domains, only desire and lubrication were the phases of the sexual cycle that explained poorer sexual functioning. Our findings suggest that obesity may be a risk factor for poor sexual function among women with GDM. Obesity in pregnancy has been associated with lower quality of life, especially in the physical and mental domains [32], [33], with daytime sleepiness [33] and with higher levels of anxiety [30] and depressive symptoms [30], [31]. These findings can help to explain the lower sexual function of our obese GDM patients compared to our normal weight GDM patients.

The finding that overweight GDM patients are at a higher risk for sexual dysfunction can have implications for practice. The team of health professionals caring for these patients in our setting is discussing the possibility of creating specific interventions for them, including multidisciplinary group counseling to discuss obesity and changes in the couple's sexual life during pregnancy. This type of intervention has not been specifically tested in obese women but has shown promising results in pregnant women in general. According to an Iranian study, after participating in four weekly sexual education classes, the mean FSFI scores of 41 healthy pregnant women went from 22.6 (±7.9) to 26.6 (±4.3), p = 0.0001 [45].

A strong point of our study was the use of the FSFI questionnaire, a validated instrument to assess female sexual function [39], which has been extensively used in studies involving pregnant women [8], [15], [22], [42], [44], [46]. An additional strong point was that our participants were homogeneous in relation to other variables, besides BMI, which could affect female sexual function [47].

We acknowledge that use of additional questionnaires to evaluate mental symptoms and quality of life of the participants could have enriched our study. Finally, our findings cannot be generalized to GDM patients of other socioeconomic strata or cultural contexts.

## Conclusion

Overweight GDM patients in the third trimester of pregnancy have lower female sexual function scores than their normal weight counterparts. Desire and lubrication were the phases of the sexual cycle with the lowest scores among overweight women. More studies, involving women of other social and cultural contexts are needed to confirm these findings.

## Supporting Information

Consent S1 **Informed consent.**
(DOCX)

Protocol S1 **Study Protocol.**
(DOCX)
