# Supplementary material for: Female Sexual Function of Overweight Women with Gestational Diabetes Mellitus – A Cross-Sectional Study
Source: PLoS One. 2014 Apr 15;9(4):e95094. doi: 10.1371/journal.pone.0095094 (PMC3988167; doi:10.1371/journal.pone.0095094)
Supplement: Consent S1 — Informed consent. (DOCX) [file pone.0095094.s001.docx]

Informed Consent S1 (translation by the author)

SÃO PAULO FEDERAL UNIVERSITY - Medical School

I _______________________________________________________ have been informed that there are some questions and doubts regarding the sexual function of women with Gestational Diabetes Mellitus who have different body weights. To help clarify these questions, researchers are conducting a study with women who have Gestational Diabetes Mellitus and have different body weights. I have been informed that Meireluci Costa Ribeiro is the main investigator of this study.

I was invited to participate in this study which involves filling a form that will collect personal information such as my age, number of pregnancies, gestational age, etc. and also a questionnaire about sexuality with 19 questions. I have been informed that it will take me approximately 10 minutes to fill these two questionnaires and that all my answers will be kept confidential and anonymous. After answering and returning the filled the questionnaires, the investigator will be available to answer any questions or doubts that I may have about sexual activity during pregnancy. My participation in the study does not involve any risks or discomforts.

I am aware that my participation is voluntary and that I have the right to refuse to answer any of the questions and to abandon the study at any time without any consequences to my antenatal care or other benefits to which I am entitled as a patient in this institution. I have also been informed that I have the right to be informed about any additional details, as well as the results of this study, at any time.

I am aware that at any time I can contact Meireluci Costa Ribeiro at Rua Estado de Israel, 639, phone 99304-1265 for more information. In case I wish to, I can also contact the institutional ethics´ committee at Rua Botucatu, 572 – 1º. andar – cjto. 14 – Telefone 5571-1062, Fax 5539-7162 – e-mail: [cepunifesp@emp.br](mailto:cepunifesp@emp.br) , to obtain more details or clarify any doubts that I may have.

I have discussed with Meireluci my decision to participate in this study. The objectives and procedures involved in this study are clear to me, as well as my rights to confidentiality and to obtain more information at any time. I also know that I will not spend or receive any money to participate in this study and that my participation will not in any way affect my antenatal care.

I voluntarily accept to participate in this study.

x __________________________________________________________

I declare that I provided all the aforementioned information to the participant and that I obtained her informed consent voluntarily and appropriately.

_________________________________ Date ___/___/___

Responsible for the study
