# Supplementary material for: Female Sexual Function of Overweight Women with Gestational Diabetes Mellitus – A Cross-Sectional Study
Source: PLoS One. 2014 Apr 15;9(4):e95094. doi: 10.1371/journal.pone.0095094 (PMC3988167; doi:10.1371/journal.pone.0095094)
Supplement: Protocol S1 — Study Protocol. (DOCX) [file pone.0095094.s002.docx]

**Study Protocol S2**


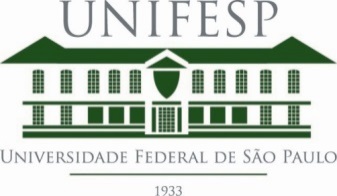


*Universidade Federal de São Paulo*

*Escola Paulista de Medicina*

Researcher: MEIRELUCI COSTA RIBEIRO

Co-Researcher: Rosiane Mattar

Obstetrics Department, São Paulo Federal University, São Paulo, Brazil

Summary of study protocol submitted to the Ethics´ Committee (translation by authors)

**Title:** Female sexual function of overweight pregnant women with Gestational Diabetes Mellitus after 28 weeks of gestation

**Main characteristic of the survey:** Cross-sectional study.

**Additional risks to the patient:** no risks, minimal discomfort, no invasive procedures

**Objectives**: To analyze the sexual function of pregnant women with Gestational Diabetes Mellitus (GDM), after 28 weeks of pregnancy, and to compare the sexual function of normal weight versus overweight women.

**Methods:** Pregnant women with GDM, in the third trimester of pregnancy, >20 years old, and managed at Centro de Diabetes of São Paulo Federal University – UNIFESP will be invited to participate. The volunteers will answer questionnaires to collect socio- demographic data and also the Female Sexual Function Index (FSFI). The results will be compared between the groups (normal weight vs overweight women with GDM). Sample size calculation was based on the 59% prevalence of sexual dysfunction symptoms among adult Brazilian women with GDM in the third trimester of pregnancy; assuming that overweight women would have a 40% higher prevalence of this disorder, with an α=0.05 and a β=90%, the study will need to recruit 62 participants in each group (normal and overweight). Data will be analyzed using the Chi-square and Student’s *t* tests to compare categorical and continuous variables between the two groups. P < 0.05 will be considered significant.
